# Dietary Selenium Levels Affect Selenoprotein Expression and Support the Interferon-γ and IL-6 Immune Response Pathways in Mice

**DOI:** 10.3390/nu7085297

**Published:** 2015-08-06

**Authors:** Petra A. Tsuji, Bradley A. Carlson, Christine B. Anderson, Harold E. Seifried, Dolph L. Hatfield, Michael T. Howard

**Affiliations:** 1 Department of Biological Sciences, Towson University, Towson, MD 21252, USA; E-Mail: ptsuji@towson.edu; 2 Molecular Biology of Selenium Section, Mouse Cancer Genetics Program, Center for Cancer Research, National Institutes of Health, Bethesda, MD 20892, USA; E-Mails: carlsonb@mail.nih.gov (B.A.C.); hatfield@mail.nih.gov (D.L.H.); 3 Human Genetics, University of Utah, Salt Lake City, UT 84112, USA; E-Mail: anderson@genetics.utah.edu; 4 Nutritional Science Research Group, National Cancer Institute, Rockville, MD 20892, USA; E-Mail: seifrieh@mail.nih.gov

**Keywords:** IL-6, interferon-γ, ribosome profiling, selenium, selenocysteine, selenoprotein

## Abstract

Selenium is an essential element that is required to support a number of cellular functions and biochemical pathways. The objective of this study was to examine the effects of reduced dietary selenium levels on gene expression to assess changes in expression of non-selenoprotein genes that may contribute to the physiological consequences of selenium deficiency. Mice were fed diets that were either deficient in selenium or supplemented with selenium in the form of sodium selenite for six weeks. Differences in liver mRNA expression and translation were measured using a combination of ribosome profiling, RNA-Seq, microarrays, and qPCR. Expression levels and translation of mRNAs encoding stress-related selenoproteins were shown to be up-regulated by increased selenium status, as were genes involved in inflammation and response to interferon-γ. Changes in serum cytokine levels were measured which confirmed that interferon-γ, as well as IL-6, were increased in selenium adequate mice. Finally, microarray and qPCR analysis of lung tissue demonstrated that the selenium effects on immune function are not limited to liver. These data are consistent with previous reports indicating that adequate selenium levels can support beneficial immune responses, and further identify the IL-6 and interferon-γ pathways as being responsive to dietary selenium intake.

## 1. Introduction

Selenium (Se) is an essential trace element that is attained through consumption of a wide variety of dietary components [1]. While cases of toxicity or extreme deficiency are rare in humans, less overt changes in dietary Se levels have been shown to significantly affect human health [2,3]. The effects ascribed to marginal changes in dietary Se are multifaceted and include supporting cardiovascular health, thyroid hormone metabolism, inflammation and immune function, as well as protection against neurodegeneration, cancer, and viral infection.

Se is incorporated into selenoproteins in the form of the amino acid selenocysteine (Sec), and the biological effects of Se are exerted primarily through the function of different selenoproteins. Defined by the incorporation of Sec and broadly classified as antioxidants, selenoproteins exhibit tissue and cell-specific expression patterns, act on a variety of substrates, and have multiple functions [3]. Among these are control of the cellular redox state and protection from oxidative damage and stress. Selenoproteins also have roles in thyroid hormone metabolism [4], intracellular Ca^++^ mobilization [5,6,7], protein folding [8], Se transport [9], and they can even catalyze intermediates in the synthesis of Sec [10,11].

The incorporation of Se into selenoproteins occurs through a complex Sec biosynthesis pathway in which this amino acid is synthesized on a dedicated tRNA (tRNA^[Ser]Sec^) [11]. The Sec moiety is then transferred to the nascent selenoprotein from Sec-tRNA^[Ser]Sec^ by decoding a UGA codon within the selenoprotein mRNA [12,13]. Se status impacts the efficiency of Sec biosynthesis [14], Um34 methylation of Sec-tRNA^[Ser]Sec^ [15,16], redefinition of the UGA codon for Sec incorporation [17], and the abundance of selenoprotein mRNA [17,18,19,20]. Under conditions of moderate Se-deficiency, it has been shown that nonessential selenoproteins involved in stress-related processes (e.g., glutathione peroxidase (Gpx) 1 and selenoprotein W (Sepw1)) are preferentially lost, whereas expression of the essential housekeeping selenoproteins (e.g., Gpx4 and thioredoxin reductase (Txnrd) 1) is preserved [21,22,23,24]. Likewise, available Se is not equally supplied to all tissues [25,26]. Under conditions of Se-deficiency, brain, testes and thyroid maintain near normal Se levels, whereas other tissues, such as liver, lung and those of the immune system, exhibit a significant decline in Se and selenoprotein synthesis.

The consequences on human health may vary greatly depending on the specific biochemical, cellular, and tissue functions that are most affected by the loss of selenoproteins in combination with interacting environmental factors such as exposure to chemical insult or viral infection. For example, several studies have found an association between Se-deficiency and an increased susceptibility to viral infection and progression of pathogenesis [27]. Studies into the mechanism by which coxsackie-viral infection, the purported etiological agent of Keshan’s disease, which is affected by Se-deficiency, suggest that the resulting changes in oxidative stress levels, and perhaps the host immune response, allow for the selection of viruses with more virulent phenotypes [28,29]. Se-deficiency has also been shown to increase the pathology of influenza viral infections in animal models [30,31], and a rare study in humans revealed that Se supplements increased the cellular immune response to poliovirus vaccination through increased production of cytokines, T-cell proliferation, and more rapid viral clearance [32].

Multiple experimental studies in animal models have revealed that Se-deficiency impairs immune response to infection, cancer, and other stimuli. Examples include a reduction in CD4+ T-cell response in Se-deficient mice challenged with a peptide/adjuvant [33], increased tumor growth and spread in a mouse model of breast cancer [34], enhanced type I allergic response in a mouse model of active cutaneous anaphylaxis [35], and increased immunotoxicity resulting from arsenic exposure [36]. In each case, the levels of interferon-γ and other cytokines were reduced in Se-deficient mice. Additional studies in mouse knockouts have demonstrated that the selenoproteome [37,38,39], as well as individual selenoproteins such as Selk [7] and Sep15 [40], appear to play direct roles in supporting immune function. While additional research is needed, the important roles that selenoproteins play in regulating cellular oxidation states, catalyzing redox reactions with protein and chemical substrates, Ca^++^ signaling, protein folding, and the downstream effects of these functions will likely be identified as the molecular mechanisms to explain the importance of dietary Se in supporting the immune systems [41], as well as other health-related physiological functions.

To fully understand the health effects of Se, it is important to identify both the changes in selenoprotein expression levels as well as changes to downstream targets that contribute to the health consequences of Se-deficiency. In this study, we have applied microarrays, RNA-Seq, qPCR, and ribosome profiling to examine the genome-wide consequences of Se-deficiency in mice. As expected from previous studies, RNA-Seq and microarray analysis identified a subset of selenoprotein genes (e.g., *Gpx1*, selenoprotein H (*Selh*), *Sepw1* and selenoprotein P (*Sepp1*)) in which mRNA abundance was significantly reduced, while the expression of other essential selenoproteins (e.g., *Gpx4*, selenophosphate synthetase 2 (*Sephs2*), and *Txnrd1*) was preserved. Ribosome profiling further indicated that translation of non-essential selenoprotein mRNAs, and particularly Sec incorporation efficiency, was also significantly affected by the availability of Se. Pathway and gene ontology analysis of genes showing increased expression in the liver and lung of Se-replete mice identified a number of inflammation-related genes that are regulated by interferon-γ. This result was confirmed by serum cytokine analyses, which showed that Se-replete mice have increased levels of circulating interleukin (IL)-6 and interferon-γ.

Although excessive releases of IL-6 and interferon-γ have been associated with inflammatory and autoimmune diseases, moderate increases of these cytokines, which are known for both their pro-inflammatory and anti-inflammatory properties, play a pivotal role in host defense through the ability to activate macrophage cell functions with important implications in health and disease states [42]. Thus, our results support previous studies suggesting that adequate Se levels are required to support a healthy and robust immune response; much of it may be mediated by interferon-γ-regulation.

## 2. Experimental Section

### 2.1. Accession Codes

Microarray and ribosome profiling data are accessible through the Gene Expression Omnibus database, accession #GSE70160 [43].

### 2.2. Materials

TRIzol reagent was purchased from Invitrogen (Carlsbad, CA, USA), iScript cDNA Synthesis kit and SYBR Green Supermix from Bio-Rad Laboratories (Hercules, CA, USA). Real-time qPCR primers were from Sigma-Genosys (St. Louis, MO, USA). Se-deficient (0 ppm sodium selenite; TD.04484) and Se-supplemented diets (0.1 ppm sodium selenite; TD.10645) were obtained from Harlan-Teklad Laboratories, Inc. (Madison, WI, USA). RNase I and RNase Inhibitor were obtained from Invitrogen/Thermo Fisher Scientific (Waltham, MA, USA). Antarctic Phosphatase was obtained from New England Biolabs (Ipswich, MA, USA). The TruSeq Small RNA Sample Prep Kit, TruSeq Stranded Total RNA Sample Preparation Kit, and Ribo-Zero Human/Mouse/Rat were obtained from Illumina (San Diego, CA, USA). Other reagents were of the highest commercially-available quality.

### 2.3. Mice, Diets, Tissue Preparations and Selenium Concentrations

This study was carried out in strict accordance with the recommendations in the Guide for the Care and Use of Laboratory Animals of the National Institutes of Health. The protocol was approved by the NCI-Bethesda Animal Care and Use Committee at the National Institutes of Health (permit number for this study is BRL-002). Upon weaning, three week-old male wild-type mice in a FVB/N background were given Se-deficient diets supplemented with 0 ppm or 0.1 ppm Se (sodium selenite) and maintained on the diets for 6 weeks prior to euthanasia via CO_2_ asphyxiation. Livers and lungs were rapidly excised, washed in PBS and immediately frozen in liquid nitrogen. Selenium concentrations in the torula yeast-based diets yeast-based diets given to animals were determined fluorometrically by the South Dakota Agricultural Laboratories (Brookings, SD, USA). Selenium concentrations were also determined in liver, lung and plasma tissues (*N* = 3 for each tissue) by this laboratory.

### 2.4. mRNA Analysis from Mouse Liver and Lung Tissue

Total RNA was isolated from tissues using TRIzol reagent following the manufacturer’s recommendation. 500 ng of total RNA were reverse transcribed using iScript cDNA synthesis kit. qPCR was performed in triplicate using iTaq Universal SYBR Green Supermix according to the manufacturer’s instructions. Primer sequences used are shown in Table S1.

### 2.5. Microarray Analysis

Total mRNA was isolated from mouse liver and lung tissues with the phenol-chloroform extraction method using TRIzol. Microarray analysis was performed on Affymetrix (Cleveland, OH, USA) Mouse 430_2.0A gene chips. Three arrays were analyzed from three different mRNA samples per Se diet. The MAS5 statistical algorithm, which normalizes each array independently and sequentially, was used for background and noise correction in the conversion of probe level to gene expression data, and absent calls were excluded from analysis. Mice on deficient dietary Se and controls (adequate Se diet) were compared by *t*-test.

### 2.6. Ribosomal Profiling

Livers were rapidly excised, and frozen in liquid nitrogen. Approximately 100 mg of tissue were pulverized by rapid agitation in a Mini-Beadbeater-8 (Biospec Products Inc., Bartlesville, OK, USA) in 1.5 mL pre-chilled lysis buffer (10 mM Tris-Cl (pH 7.5), 300 mM KCl, 10 mM MgCl_2_, 200 μg/mL cycloheximide (Sigma-Aldrich, MO, USA), 1 mM DTT, and 1% Triton X-100). 1 mL of crude lysate was incubated with 500 units of RNase I for 30 min at 25 °C. Monosomes were isolated by centrifugation through a sucrose cushion at 48,000 RPM (100,000 max. rcf) for 3 h in a TL100 ultracentrifuge. Ribonuclease-resistant RNA fragments were isolated from the pellet by TRIzol extraction and electrophoresis on a 15% polyacrylamide, 8 M urea gel. The region of the gel containing ~26–36 nt size RNA fragments was excised, and RNA isolated by passive elution prior to library construction.

Gel purified ribosome footprints were treated with 5 units of Antarctic Phosphatase in the presence of 40 units of RNase inhibitor for 30 min at 37 °C followed by 5 min at 65 °C to deactivate the enzyme. Small RNA sequencing libraries were constructed using the Illumina TruSeq Small RNA Sample Prep Kit (Illumina, San Diego, CA, USA) according to the manufacturer’s instructions. Following limited PCR amplification (11 cycles), the PCR-amplified library was resolved on a 6% Novex TBE PAGE gel (Invitrogen/Thermo Fisher Scientific, Grand Island, NY, USA) and a gel fragment representing the size range expected for ligation of ribosome footprints was excised from the gel. Small RNA library molecules were eluted by soaking the crushed gel fragment overnight in ultra-pure water at room temperature. Libraries were subjected to 50 cycle single-end sequencing on an Illumina HiSeq 2000 Instrument.

### 2.7. RNA-Sequencing Analysis (RNA-Seq)

Livers were rapidly excised, and frozen in liquid nitrogen. Approximately 100 mg of tissue were pulverized under liquid nitrogen by mortar and pestle. While still frozen, 2 mL of TRIzol were added and total RNA isolated according to the manufacturer’s instructions. Library construction was performed using the Illumina TruSeq Stranded Total RNA Sample Preparation Kit with Ribo-Zero Human/Mouse/Rat (Illumina, San Diego, CA, USA). Briefly, ribosomal RNA was removed from total RNA samples using biotinylated Ribo-Zero oligos attached to magnetic beads. Following purification, the rRNA-depleted sample was fragmented and primed with random hexamers. First strand reverse transcription was accomplished using Superscript II Reverse Transcriptase (Invitrogen/Thermo Fisher Scientific, Grand Island, NY, USA). Second strand cDNA synthesis was accomplished using DNA polymerase I and RNase H under conditions in which dUTP is substituted for dTTP. An A-base was added to the blunt ends and ligated to adapters containing a T-base overhang. Ligated fragments were PCR-amplified (12–15 cycles) under conditions in which the PCR reaction enables amplification of the first strand cDNA product only. Libraries were subjected to 50-cycle single-end sequencing on an Illumina HiSeq 2000 Instrument.

### 2.8. Bioinformatic Analysis of RNA-Seq and Ribosome Profiling

Adapter sequences were removed using the Hannon laboratory FastX toolkit [44] rRNA sequences were removed using Bowtie (Johns Hopkins University, Baltimore, MD, USA) [45]. Sequences were aligned against mouse rRNA, and all unaligned sequences were retained for further processing. RefSeq FASTA sequences were obtained from the UCSC genome browser (mm9). These FASTA files were reduced to a single entry for each mRNA corresponding to the longest isoform. For coding sequence alignments, the first 15 and last 3 codons were excluded to avoid bias at the initiation and termination codons. Uniquely aligning sequences, allowing for 2 mismatches, were identified using the Bowtie sequence aligner [45].

Ribosome profiling and total RNA sequences were aligned separately to selenoprotein mRNAs using the Bowtie alignment parameters described above against the following RefSeq entries: NM_027652.2 *Seli*, NM_027905 *Selo*, NM_007860.3 *Dio1*, NM_013759.2 *Sepx1*, NM_024439.3 *Sels*, NM_015762.2 *Txnrd1*, NM_008160.6 *Gpx1*, NM_008162.2 *Gpx4*, NM_009155.3 *Sepp1*, NM_053102.2 *Sep15*, NM_013711.3 *Txnrd2*, NM_019979.2 *Selk*, NM_001040396.2 *Selt*, NM_009156.2 *Sepw1*, NM_009266.3 *Sephs2*, NM_030677.2 *Gpx2*, NM_001178058.1 *Txnrd3*, NM_029100.2 *Sepn1*, NM_053267.2 *Selm*, NM_008161.3 *Gpx3*, NM_001033166.2 *Selh*, NM_172119.2 *Dio3*, NM_010050.2 *Dio2*, NM_175033.3 *Selv*, NM_008084.2 *Gapdh*.

For quantitative analysis, the 5′ end of ribosome profiling and RNA reads were offset to the predicted A-site, as determined previously [17]. Total mapped reads used to derive RPKM calculations (reads per kilobase per total million mapped reads) were determined by aligning sequences to the mRNAs of the RefSeq database described above. Analysis of ribosome footprinting upstream of the UGA-Sec codon excluded the first 15 codons after the initiation codon and the 5 codons preceding the UGA-Sec codon. Ribosome footprint RPKMs downstream of the UGA-Sec were calculated from the second codon following UGA-Sec to the third codon preceding the stop codon. All offset, quantitative, and phasing analyses for ribosome profiling data were conducted using Bowtie alignments and custom perl scripts (MTH).

Genome-wide changes in mRNA abundance or ribosome protection were determined using the edgeR package of Bioconductor [46]. mRNA or ribosome protected fragments showing a >1.5-fold change in abundance and a false discovery rate of <0.05 were considered to be differentially expressed.

### 2.9. Ingenuity Pathway Analysis

Gene expression results from microarrays, RNA-Seq, and ribosomal profiling experiments that were significantly different between mice with adequate Se *versus* deficient Se levels (*p* < 0.05 for microarrays, 1.5-fold change and false discovery rate (FDR) <0.05 for RNA-Seq and ribosome profiling) were subjected to Ingenuity Pathway Analysis (IPA (Qiagen, Valencia, CA, USA)) to elucidate common networks of gene expression according to functional biological processes.

### 2.10. Cytokine Analyses

Mouse blood was collected by cardiac puncture (*N* = 5/diet), centrifuged at 3000 rcf for 5 min in heparinized tubes, and serum was snap-frozen and stored at −80 °C. Using the mouse TH1/TH2 7-Plex assay kit, serum protein levels of interferon-γ, interleukin-1β, interleukin-6, KC/GRO (Cxcl1), interleukin-10, interleukin-12p70 and tumor necrosis factor-α were assessed in a sandwich immunoassay format using a SECTOR^®^ Imager 2400 according to the manufacturer’s instructions (MesoScale Discovery, Gaithersburg, MD, USA). An eight-point standard curve was used to calculate the concentration of analytes in the serum samples. Samples and standards were assayed in duplicates.

### 2.11. Statistical Analyses

Serum cytokine levels, tissue selenium levels, and qPCR data for mRNA expression are presented as the mean ± SE, and were analyzed by Student’s t-test with GraphPad Prism (version 5, La Jolla, CA, USA). The level of significance was set at *p* = 0.05, and statistically significant differences compared to controls were indicated in figures as follows: * *p* < 0.05, ** *p* < 0.01, *** *p* < 0.001. Selenoprotein mRNA and 3′ RPKM values were determined from two biological replicas and reported as the mean and standard deviation for each selenoprotein. Correlations between the replicas were further analyzed by plotting edgeR normalized CPM (counts per million) values for all RefSeq mRNAs in each biological replicate against the other (Figure S1). *R*^2^ values were greater than 0.99 for both the 0 Se and 0.1 Se replicate samples. EdgeR package from Bioconductor was used to determine differentially expressed genes in RNA-Seq and ribosome profiling experiments for all genes with >1 count per million mapped reads in at least 3 samples. Genes with a greater than 1.5-fold change and a FDR of <0.05 were considered to be differentially expressed.

## 3. Results

**Figure 1:**
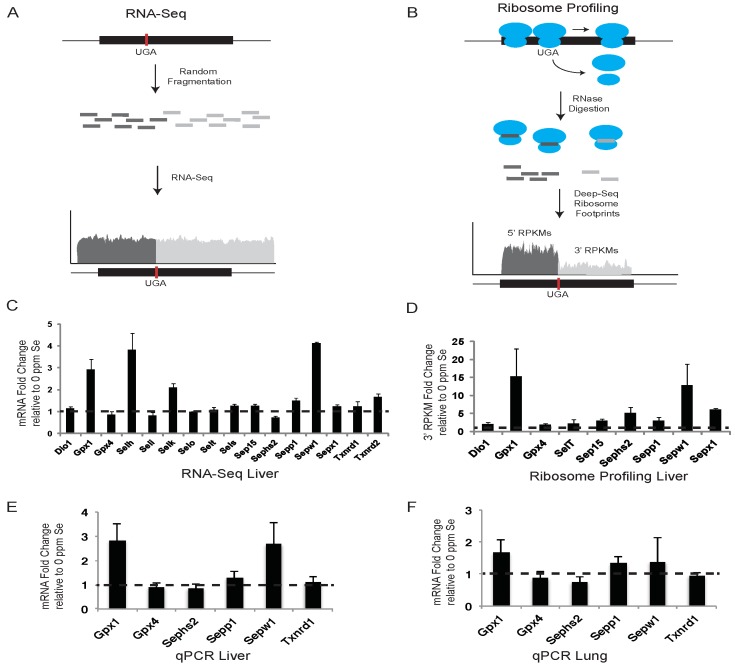
RNA-Seq and ribosome profiling of selenoprotein mRNAs. (**A**) and (**B**) show conceptual illustrations of RNA-Seq and ribosome profiling experiments, respectively. A representative selenoprotein mRNA is shown with the position of the UGA-Sec codon indicated by the vertical red bar. Randomly sheared (**A**) or ribosome protected (**B**) mRNA fragments are shown in dark or light grey to indicate whether they are positioned 5′ or 3′ of the UGA-Sec codon. (**C**) The histogram shows the fold-change in liver sequence reads per kilobase per million mapped reads (RPKM) in mice fed diets supplemented with 0.1 ppm Se (0.1 Se) relative to those fed unsupplemented diets (0 ppm Se). Dashed line across the histogram indicates a fold change value of 1 (no difference). (**D**–**F**) Same as in (**C**) for liver ribosome protected fragments, Liver qPCR and lung qPCR, respectively, with the exception that in (**D**) the RPKM values were determined only for the portion of the selenoprotein mRNA located 3′ of the UGA-Sec codon. Standard deviations were calculated by the Propagation of Error method.

We have applied complimentary genome-wide methods, RNA-Seq and ribosome profiling as shown schematically in Figure 1A,B, respectively, and microarrays (see below), as well as qPCR to determine changes in gene expression and translational activity in liver and lung tissues of mice fed Se-deficient or Se-adequate diets. Liver was selected, as it is where Se is processed and converted into other forms appropriate for delivery through the circulatory system to other tissues, or for excretion [47]. Delivery of Se to other tissues is primarily mediated by uptake from the blood stream of liver-produced Sepp1, which contains up to 10 Se atoms in the form of Sec residues. Lung was selected as a second tissue, because selenoprotein expression in this tissue is strongly affected by Se dietary intake, and due to the fact that Se status has been proposed as a risk factor and modulation of intake as a potential preventative for a number of lung disorders with involvement of oxidative stress and inflammation [48,49,50,51].

Selenium concentrations of diets provided to animals were 0.0238 ppm for the selenium-deficient diet (“0 Se”) and 0.128 ppm for the selenium-adequate diet (“0.1 Se”). Selenium concentrations of liver, lung and plasma samples were: mean (± SEM) for liver, 0.1065 ± 0.0143 μg/g and 1.0830 ± 0.0240 μg/g; mean for lung, 0.1096 ± 0.0129 μg/g and 0.3163 ± 0.0149 μg/g; and mean for plasma, 0.0559 ± 0.0090 μg/mL and 0.3510 ± 0.0078 μg/mL in animals maintained on 0 Se and 0.1 Se diets, respectively. Thus, tissue selenium levels were up to ~10 fold lower in mice maintained on a selenium-deficient diet (e.g., liver: *p* < 0.0001).

### 3.1. RNA-Seq and qPCR Analyses of Selenoprotein mRNA Expression

The effects of altered dietary Se intake on hepatic selenoprotein mRNA levels were examined using RNA-Seq and qPCR. Liver mRNA was isolated from mice fed Se-deficient or -adequate diets and prepared for deep sequencing or qPCR as described in the Experimental Section. For RNA-Seq, deep-sequence reads were aligned against RefSeq selenoprotein mRNAs, quantified as reads per kilobase per million mapped reads (RPKM), and presented as the fold changes in expression between mice fed diets supplemented with 0.1 ppm Se and 0 ppm Se (Figure 1C). Selenoprotein expression was also examined by qPCR in liver (Figure 1E) and lung tissue (Figure 1F) for a subset of selenoproteins in Se-replete mice, and expressed as the fold change in expression relative to mice fed diets with no Se supplementation.

RNA-Seq data revealed that liver mRNA levels for *Gpx1*, *Selh*, *Selk*, and *Sepw1*, showed the greatest response to Se availability with Se-adequate mice having between 2- and 4-fold more mRNA than Se-deficient mice. *Sepp1* and *Txnrd2* mRNAs had an intermediate response with ~1.5-fold increase, whereas other selenoproteins revealed less than a 1.5-fold change in mRNA abundance. qPCR analysis was in close agreement with the RNA-Seq results for the selenoproteins examined (Figure 1E). As expected, mouse liver mRNA levels of stress-related selenoproteins known to be sensitive to dietary Se concentrations (e.g., *Gpx1*, *Sepp1*, *Sepw1*) [21,22] were shown by qPCR to be significantly increased in mice maintained on a Se-adequate diet compared to mice fed Se-deficient chow. Similarly, selenoprotein mRNA levels of *Gpx1* and *Sepp1* were also significantly (*p* < 0.05) elevated in lung tissues of mice maintained on a Se-adequate diet, whereas *Sepw1* mRNA was only modestly increased (*p* > 0.05) (Figure 1F). Mouse mRNA levels of house-keeping selenoproteins, which are known to be more resistant to dietary Se changes (e.g., *Txnrd1*, *Gpx4*, and *Sephs2*) [21,22], did not significantly change between Se-deficient and Se-adequate diets in liver or lung tissue (Figure 1E,F).

### 3.2. Ribosome Profiling Analysis of Dietary Se Effects on Selenoprotein mRNA Translation

**Figure 2:**
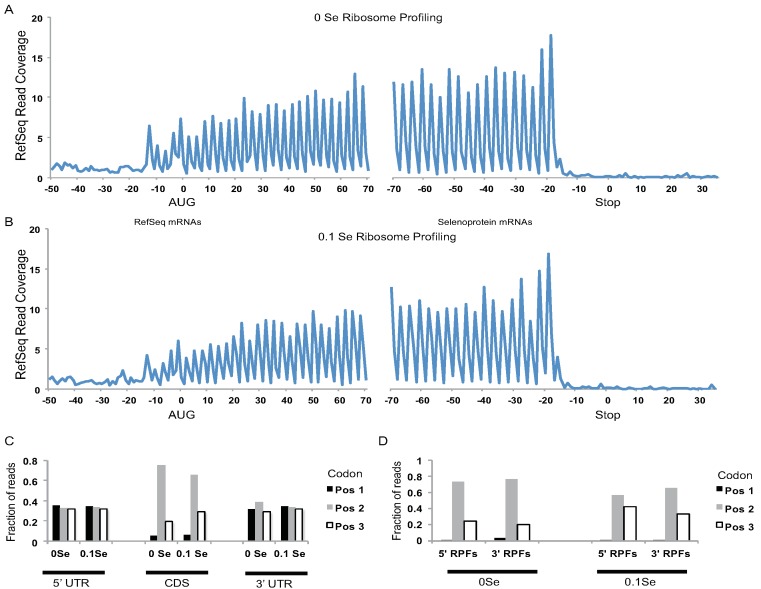
Analyses of ribosome footprint (RPF) distribution on RefSeq and selenoprotein mRNAs. (**A**) 0 ppm Se and (**B**) 0.1 ppm Se samples. RPF 5′ ends were aligned to all RefSeq mRNAs with coding DNA sequences (CDSs) > 400 nts and their positions relative to the start codon (0 position, left hand side) and stop codon (0 position, right hand side) were determined. The fraction of RPF 5′ ends aligning to nts −50 to +70, relative to RefSeq start codons, and nts −70 to +35, relative to RefSeq stop codons (0 position, right hand side), are shown. Distinct 3nt periodicity can be seen starting 13 nts upstream of start codons and ending 16 nts upstream of stop codons indicating that the first nucleotide of the A-site codon is located ~16 nts downstream of the 5′ end of the RPFs. Y-axis represents the fraction of reads covering each nucleotide summed for all aligned RefSeq mRNAs (CDS > 400 nts). (**C**) The fraction of RPFs with 5′ ends mapping to the first, second, or third codon position for CDSs and UTRs of RefSeq mRNAs. For UTRs, the first, second, and third “codon” position were determined relative to the CDS reading frame. (**D**) The 5′ end of RPFs from the 0 ppm Se or 0.1 ppm Se samples were aligned to selenoprotein mRNAs. The fraction of RPF 5′ ends that aligned to codon positions 1, 2, or 3 upstream (5′ RPFs) or downstream (3′ RPFs) of the UGA-Sec codon is shown.

Assessment of ribosome footprints aligned to all RefSeq mRNAs revealed that the footprints were highly enriched in coding DNA sequences (CDS) relative to UTRs (Figure 2A,B), and were positioned with a strong triplet phasing corresponding to the expected codon step size of actively translating ribosomes (Figure 2C). Likewise, triplet phasing was observed for the ribosome footprints located upstream and downstream of UGA-Sec codons in the selenoprotein mRNAs (Figure 2D) demonstrating that the ribonuclease resistant fragments obtained from selenoprotein mRNAs have the expected features of footprints obtained from actively translating ribosomes.

Previous studies have shown that, in addition to mRNA abundance, dietary Se levels can regulate the efficiency of Sec incorporation, which is in competition with termination at the UGA-Sec codon [17]. Due to the loss of ribosomes to termination at the UGA-Sec codon, we propose that quantifying ribosome footprints located downstream of the UGA-Sec codon (3′ RPKMs) provides the most accurate measure of translational activity leading to the production of full-length selenoproteins. The fold-changes in 3′ RPKMs between Se-adequate and Se-deficient mice are shown in Figure 1D. In some selenoproteins, the UGA-Sec codon is located near the 3′ end of the open reading frame (ORF) preventing analysis of ribosome density downstream of the UGA-Sec codon. For the non-C-terminal Sec selenoproteins expressed in liver, the fold change in 3′ RPKMs was increased by the addition of 0.1 ppm Se to the diet for all selenoproteins. *Gpx1*, *Sepx1*, *Sepw1* and *Sephs2* were increased by 15, 13, 6, and 5-fold, respectively. All other selenoproteins analyzed had a 2-fold or greater increase in 3′ RPKMs, with the exception of the 1.9-fold increase observed for *Gpx4* (Figure 1D). These results indicate that Se affects selenoprotein synthesis to variable degrees, with the strongest effects being exerted on mRNA expression and translation of stress-related selenoproteins.

### 3.3. Global mRNA Expression Analysis (Microarray, RNA-Seq, Ribosome Profiling) and Validation (qPCR)

Microarray analyses of total mRNA isolated from mouse liver and lung tissue were performed using Affymetrix Mouse 430_2.0A gene chips of three mice per diet. Likewise, total mRNA or ribosome protected fragments were isolated from mouse liver (two mice per diet) and prepared for deep sequencing on the Illumina HiSeq 2000 platform. The number of counts uniquely aligning to each mRNA was determined and utilized for differential expression analysis using edgeR, a Bioconductor program designed to identify differential expression of genes from deep sequencing data sets [46]. The top differentially expressed genes from microarrays, RNA-Seq, and ribosome profiling are shown in Table 1. Results indicated involvement of inflammation and immune system genes, especially in mouse lung tissue, as increased interferon-γ-regulated pro-inflammatory gene expression changes were observed in mice on the Se-adequate diet compared to the Se-deficient diet. In contrast, the top down-regulated gene expression changes with microarray, RNA-seq or ribosomal profiling did not appear to be directly involved with interferon-γ signaling.

Ingenuity pathway analysis (IPA) was utilized for data generated by microarray, RNA-Seq and ribosomal profiling to narrow our focus on groups of significantly changed genes according to their biological processes in which they function. In this manner, potential direct and indirect impacts of dietary Se on specific signaling networks, and thus human diseases, may be inferred. Pathway analysis results for the microarray data from both lung and liver tissues, as well as for the RNA-Seq and ribosomal profiling data from hepatic tissue, indicated dietary Se impacts inflammatory or immune pathway signaling networks. Primarily, these pathways appeared to be regulated by interferon-γ (Figure S2). Correspondingly, mRNA coding for the interferon-γ-regulated guanylate binding proteins (Gbp), Igtp and Irgm2 were among those most highly up-regulated (Table 1).

**Figure 3:**
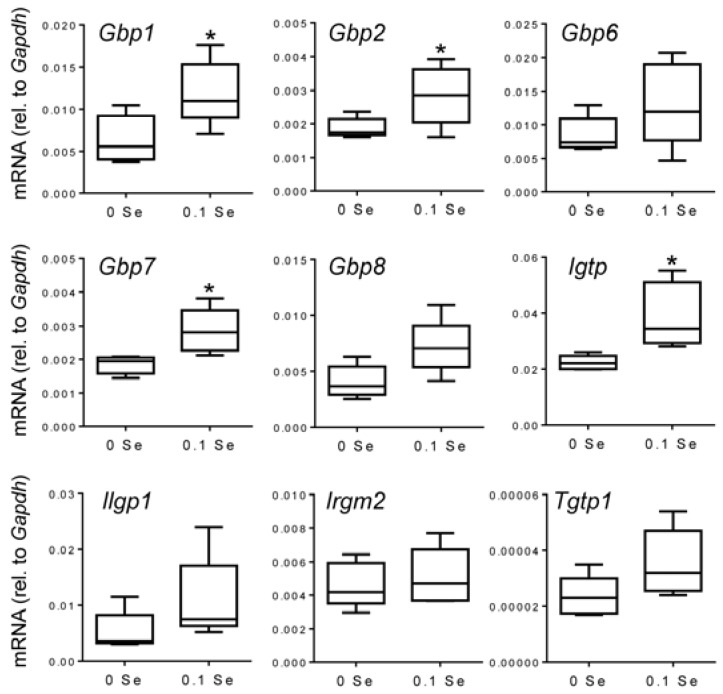
Analysis of interferon-γ-regulated pro-inflammatory mRNA expression in liver by qPCR. Liver mRNA levels for the indicated genes were measured by qPCR for mice fed 0.1 ppm and 0 ppm Se supplemented diets (*n* = 5). mRNA levels for each gene were normalized to GAPDH, and graphed with box (median plus 75th and 25th percentile) and whisker (minimum and maximum points) plots. (* *p* < 0.05 compared to 0 Se).

**Figure 4:**
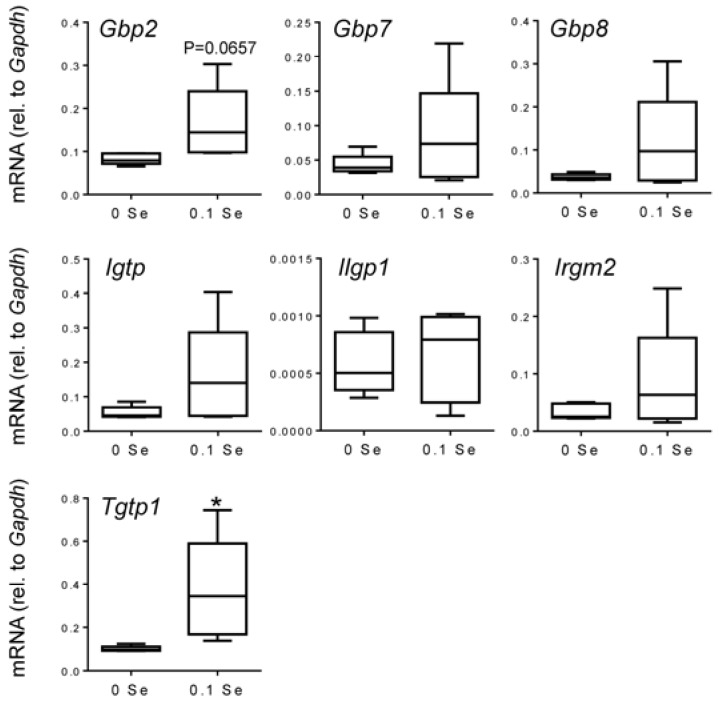
Analysis of interferon-γ-regulated pro-inflammatory mRNA expression in lung by qPCR. Lung mRNA levels for the indicated genes were measured by qPCR for mice fed 0.1 ppm and 0 ppm Se supplemented diets (*n* = 5). mRNA levels for each gene were normalized to GAPDH, and graphed with box (median plus 75th and 25th percentile) and whisker (minimum and maximum points) plots. (* *p* < 0.05 compared to 0 Se).

Many of these cytokine responsive gene changes were subsequently validated with real-time quantitative reverse-transcriptase PCR (qPCR). *Gbp1*, *2*, *6*, *7* and *8* were increased in both liver and lung tissues of mice on the Se-adequate diet as shown in Figure 3 and Figure 4, and statistical significance was observed for most, as indicated. Other interferon-γ/Stat-1-regulated genes, such as *Irgm2* (*p* = 0.06), *Igtp* (*p* < 0.05), and *Tgtp1* (*p* < 0.05), were also increased in lung tissues of mice on Se-adequate diets.

### 3.4. Serum Cytokine Analyses

**Figure 5:**
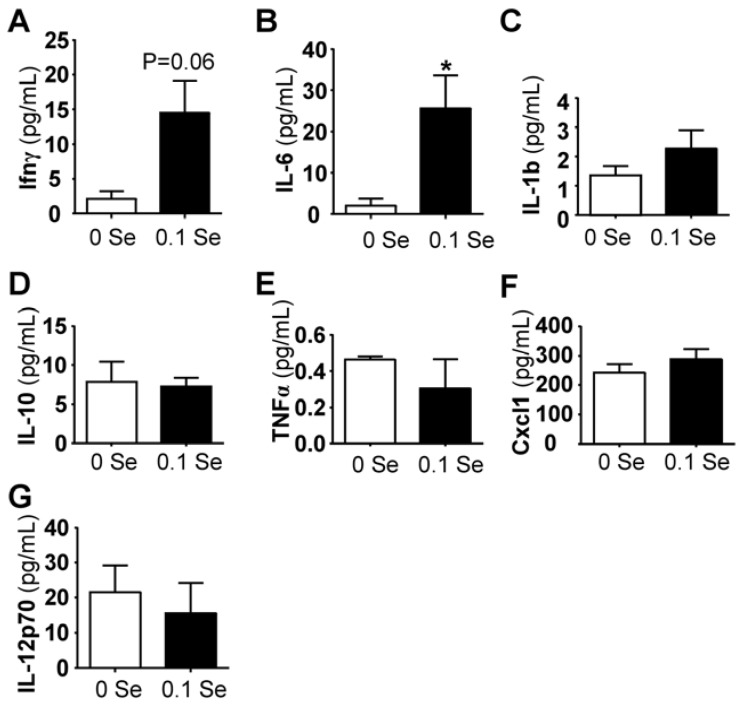
Serum cytokine analysis. Cytokine levels are expressed in pg/mL serum. The columns represent the mean (*N* = 5) and standard error of each respective cytokine in mice fed 0 ppm (unshaded bar) or 0.1 ppm Se (shaded bar) supplemented diets. (* *p* < 0.05 compared to 0 Se).

Among the pro-inflammatory serum cytokines analyzed, interferon-γ and interleukin IL-6 (*p* < 0.05) were highly increased in mice on Se-adequate diets, whereas no difference was observed for IL-1β, IL-10, IL-12p70, Cxcl1 (KC/GRO) or TNFα (Figure 5). Serum interferon-γ was increased more than 3-fold (*p* = 0.06), likely explaining the increase of many of the interferon-γ-regulated mRNA levels observed in the liver and lung tissues of these mice.

**Table 1:** Genes showing the largest fold change in mRNA levels or translation in mice fed diets supplemented with 0.1 ppm Se relative to mice fed 0 ppm Se diets.

	Liver Microarray		Lung Microarray		Liver RPF		Liver RNA-seq	
	Gene	Fold Change	Gene	Fold Change	Gene	Fold Change	Gene	Fold Change Adjusted
**up-regulated**	*Sepw1*	3.455	*Gbp8*	3.793	*Sepw1*	21.139	*Gm4841*	7.463
	*Cyp2b6*	2.600	*Tgtp1*	3.494	*SelH*	14.186	*Lcn2*	6.548
	*Rilpl1*	2.183	*Serpina3g*	3.415	*Gm4841*	6.781	*Lmod2*	6.524
	*C11orf31*	2.165	*Igtp*	3.311	*Zbtb16*	6.321	*Bhlha15*	5.322
	*Igtp*	2.164	*Iigp1*	3.290	*Saa1*	5.150	*Saa1*	5.294
	*p15*	2.146	*Irgm2*	3.204	*Saa2*	4.517	*Saa2*	5.151
	*Cxcl9*	2.088	*Gbp6*	3.073	*Gbp10*	4.244	*Sepw1*	4.274
	*Acacb*	2.046	*Ms4a4c*	2.839	*Pus3*	3.333	*Xlr3a*	4.258
	*Map3K4*	1.931	*Gbp7*	2.795	*Fgl1*	3.318	*Fgl1*	3.800
	*Ptpn18*	1.908	*Irgm1*	2.528	*Creld2*	3.209	*SelH*	3.794
	*Lpin2*	1.900	*Cd274*	2.502	*Gemin6*	3.195	*Derl3*	3.755
	*Trim7*	1.880	*Ifi47*	2.475	*Lpin1*	3.187	*Cxcl9*	3.562
	*Marco*	1.868	*Gbp3*	2.441	*Rgs16*	2.859	*Zbtb16*	3.552
	*Zbtb16*	1.845	*Stat1*	2.358	*Gbp3*	2.805	*Gm12250*	3.539
	*Nfil3*	1.835	*Gvin1*	2.332	*Gpx1*	2.776	*Gbp10*	3.495
	*Gbp7*	1.808	*Gbp2*	2.255	*Gbp1*	2.628	*Scara5*	3.216
	*Il1r1*	1.803	*Gzmb*	2.238	*Fkbp5*	2.557	*Ubd*	3.172
	*Fcgr4*	1.790	*Tlr13*	2.095	*Gbp2*	2.546	*Nnmt*	3.102
**down-regulated**	*Iffo2*	−2.093	*Cyp2a4*	−4.931	*Gsta2*	−4.924	*Fos*	−5.160
	*Csad*	−1.913	*Pln*	−3.448	*Cyp26a1*	−4.143	*Fst*	−3.480
	*Cyr61*	−1.819	*Areg*	−2.562	*Slc25a25*	−2.490	*Gpr64*	−3.018
	*Ank3*	−1.785	*Fam216b*	−2.268	*Pctp*	−2.479	*Gse1*	−2.901
	*Fign*	−1.778	*Ttc9*	−2.169	*Ppp1r10*	−2.337	*Cib3*	−2.792
	*Actr1a*	−1.730	*Chad*	−2.151	*Mup10*	−2.159	*Ltbp3*	−2.603
	*Snrnp48*	−1.729	*Dnah7b*	−2.088	*Tsc22d1*	−1.979	*Col1a1*	−2.522
	*Rapgef3*	−1.653	*Riiad1*	−2.002	*Hamp2*	−1.964	*Pcdh18*	−2.432
	*Pard6B*	−1.649	*Akap14*	−1.938	*G6pc*	−1.862	*Slc25a25*	−2.426
	*Pcf11*	−1.640	*Nr4a2*	−1.911	*Cyp2a5*	−1.829	*Svep1*	−2.425
	*Adora1*	−1.639	*Dnali1*	−1.887	*Sgk2*	−1.815	*Arhgap23*	−2.346
	*Whrn*	−1.614	*Ms4a1*	−1.877	*Ccrn4l*	−1.809	*Ppp1r10*	−2.323
	*Cyp2j9*	−1.612	*Dnali1*	−1.835	*Gpcpd1*	−1.750	*Cyp2d12*	−2.275
	*Itsn1*	−1.598	*Spon2*	−1.788	*Reln*	−1.711	*Bahcc1*	−2.259
	*Ank3*	−1.566	*Ccdc40*	−1.787	*Cyp2b9*	−1.704	*Sipa1l2*	−2.205
	*Ndufab1*	−1.568	*Anxa8*	−1.786	*Srsf5*	−1.695	*Ltbp4*	−2.198
	*March5*	−1.568	*Ptgs2*	−1.76	*S1pr1*	−1.691	*Fam38a*	−2.173
	*Dimt1*	−1.575	*Nmt2*	−1.758	*Adck5*	−1.654	*Hspg2*	−2.155

The top up-regulated and down-regulated genes by fold change for the liver microarray, lung microarray, liver ribosome profiling (RPF), and liver RNA-Seq data are shown. All genes had *p*-values < 0.05 (microarrays) or FDR < 0.05 (RNA-Seq, ribosome profiling).

## 4. Discussion

Reduced dietary Se intake is known to have gene-specific effects on the levels of both selenoprotein mRNA and protein. Consistent with previous reports [17], we found the mRNA levels of the stress-related selenoproteins, *Gpx1*, *Sepw1*, *Selh*, and *Selk*, appeared to be strongly affected by changes in Se status, while mRNA levels for other selenoproteins were largely unaffected. Ribosome profiling showed that in addition to mRNA levels, the efficiency of Sec incorporation, revealed by ribosome density downstream of the UGA-Sec codon, was also affected by dietary Se levels in a gene-specific manner. Here we observed that all selenoproteins amenable to this analysis have reduced translation, to variable degrees, downstream of the UGA-Sec codon when Se is limiting.

Whether health effects attributed to Se are mediated directly by changes in the abundance of specific selenoproteins, secondarily through changes in the cellular and biochemical pathways in which they act, or indirectly through non-selenoprotein mechanisms, is not known. For many selenoproteins, studies on whole animal or conditional deletions in rodent models have provided significant insight into the *in vivo* function and the phenotypic consequences of their absence [52]. The list of biochemical functions is long, including many enzymes involved in cellular redox control and protection, thyroid hormone metabolism, protein folding, Ca^++^ signaling, and Sec synthesis. In addition to other cellular pathways, the entire selenoproteome [37], as well as several individual selenoproteins in particular, have been implicated in immune system function. For example, *Selk* knockout mice exhibit defects in Ca^++^ flux that impacted T cell and neutrophil migration, Fcγ receptor-mediated oxidative burst in macrophages, and exhibit decreased viral clearance during infection [7]. Mice with *Sep15* deleted have increased interferon-γ signaling with functional consequences for induction and development of colon cancer [40]. In addition, the expression of some selenoproteins, such as *Sels*, can be modulated by inflammation and pro-inflammatory cytokines [8].

Several studies have also shown that Se can influence epigenetic modifications of DNA and histones (reviewed in [53]). Recent examples include the identification of differentially methylated and expressed inflammatory-related genes in patients with Keshan’s disease [54], and the finding that Se supplementation can control epigenetic regulation of pro-inflammatory gene expression in macrophages by affecting histone H4 acetylation [55]. In the latter study, macrophages isolated from the bone marrow of *Trsp^fl/fl^Cre^LysM^* mice suggested that selenoprotein expression was important for the observed inhibition of histone H4 acetylation. While epigenetic effects of Se on gene expression is a relatively new area of investigation with much to be learned, the data thus far suggest an additional mechanism by which changes in Se status may have broad effects on gene expression to affect the immune system and other cellular pathways.

To determine the extent to which dietary Se alters expression of non-selenoprotein genes, we measured mRNA levels and translational activity in liver and lung tissues of mice fed Se-deficient *vs.* adequate diets. Surprisingly few genes were found to be differentially expressed at 2-fold or greater cutoff, especially using microarray analyses (Table 1). Consequently, we chose to analyze all genes with greater than 1.5-fold change in expression for affected cellular pathways and functions using IPA and Gene Ontology analyses algorithms. IPA analysis revealed that dietary Se consistently impacted inflammatory or immune pathway signaling networks in both liver and lung tissues of mice. In particular, mRNA levels of interferon-γ-regulated genes, such as guanylate binding proteins, were significantly increased in Se-replete mice. The importance of interferon-γ-regulation is also reflected in the top IPA regulator effect networks constructed from microarray, RNA-Seq and ribosome profiling data (Figure S2). Using any of the three types of gene analyses, IPA predicted *infection of mammalia* to be inhibited based on Se-mediated changes in interferon-γ-regulated gene expression. In support of the IPA prediction, Gene Ontology (GO) analysis revealed a clear enrichment for immune system related GO terms with *immune system process* and *response to interferon-γ* as the top highly significant GO terms associated with each data set.

The observed enrichment for interferon-γ-responsive genes was confirmed by qPCR with significance being reached for many. We observed some variation in the extent to which the interferon-γ-responsive genes were increased in mice from the 0.1 ppm Se supplemented diet group; this variation can be observed in the box-and-whisker plots presented in Figure 2 and Figure 3. The variability of the interferon response is somewhat surprising given these mice are isogenic and were maintained under near identical conditions. One possible explanation comes from the well-known link between epigenetic variation and diet. It has been shown that even isogenic mice exposed to the same environment exhibit intrinsic epigenetic variation, and that this individual variation can be increased under conditions of sustained dietary change [56]. Additionally, because animals were housed in groups of 2–5 animals, individual responses to social stressors are difficult to account for, or exclude, and may also have contributed to observed variations. Although the basis for the individual variability even within litter mates is unclear, the observation that multiple interferon-γ-response genes are up-regulated in Se-replete mice compared to mice maintained on a Se-deficient diet was consistent and supported by the cytokine analysis discussed below. Future studies into the mechanism(s) responsible for this interferon-γ response variation are warranted as they may provide additional insights into the mechanisms driving variability of individual responses to Se intake in humans.

Measurements of serum cytokine levels in the 0 and 0.1 ppm Se supplemented diet groups revealed increased levels of interferon-γ and IL-6 in Se-replete mice. Outside the integral role of IL-6 on regulation of metabolism and regeneration of hepatic cells, both IL-6 and interferon-γ have broad effects on the cells of the immune system, and play significant roles in mounting, sustaining, or suppressing the inflammatory process [42]. The balance between cytokine levels, or the cytokine profile, determines the ability of the immune system to respond appropriately to challenges. In particular, increased interferon-γ levels have been shown to increase innate resistance to infections [57]. Thus, adequate dietary selenium may decrease susceptibility to diseases, mediated, at least in part, through interferon-γ-regulated gene expression changes.

Drugs or other approaches to inhibit or modulate cytokine activity have gained recent attention as promising new therapeutic approaches to the treatment of diseases involving chronic inflammation, such as autoimmune disorders and cancer [58,59]. Based on our results, we propose that, in addition to the effects that Se status has on selenoprotein expression, changes in Se dietary intake can have therapeutic consequences in the appropriate disease context through modulation of the immune system and control of inflammation. 

## 5. Conclusions

Dietary Se levels affect selenoprotein synthesis by altering mRNA abundance and Sec-incorporation efficiencies in a gene-specific manner with the stress-related selenoproteins being most responsive to changing Se status. In addition, adequate levels of Se are required to promote the expression of interferon-γ and IL-6, which are key mediators of immune system function and inflammation. Further studies will be required to determine if the observed effects on immune system pathways are mediated through alterations in selenoprotein synthesis, and to what extent Se-dependent changes in immune system function may account for the diversity of individual health effects attributed to dietary Se.

## Supplementary Files

Supplementary File 1
