# Supplementary material for: Dietary Selenium Levels Affect Selenoprotein Expression and Support the Interferon-γ and IL-6 Immune Response Pathways in Mice
Source: Nutrients. 2015 Aug 6;7(8):6529–49. doi: 10.3390/nu7085297 (PMC4555136; doi:10.3390/nu7085297)
Supplement: Supplementary File 1 [file nutrients-07-05297-s001.docx]

Supplementary

**Table S1.** Primers used for measuring the mRNA levels by qPCR.

| **Target gene** | **Accession No.** | **Sequence** |
| --- | --- | --- |
| *Gapdh* | NM_008084 | fwd ATGTGTCCGTCGTGGATCT |
|  |  | rev GTTGAAGTCGCAGGAGACAA |
| *Gbp1* | NM_010259 | fwd GATTTCTCCCTGGATCTGGA |
|  |  | rev CACAGGCGAGGCATATTAAA |
| *Gbp2* | NM_010260.1 | fwd TGCACAGGCCAATTGAGAGT |
|  |  | rev GCTACCAGCAGGTTCCCTAA |
| *Gbp6* | NM_194336.2 | fwd CTCTGGGACCAGGTTGCTAT |
|  |  | rev TGGATGCTTGCATTCTGGGT |
| *Gbp7* | NM_145545.4 | fwd AATCCGGTGCAGGCTGGTTA |
|  |  | rev TGCTTTGGAGTTCACCCTCAG |
| *Gbp8* | NM_029509.4 | fwd GGACCAGATTCATCTACGTTGG |
|  |  | rev ATGGCTTCATGGTTCACGGA |
| *Tgtp1* | NM_011579.3 | fwd GTCACCACTGCTGAGCTTCT |
|  |  | rev TTCAGAGATGATTTTGCTTTCCC |
| *Igtp* | NM_018738.4 | fwd ATCGCTCTTCCCCACAGGAC |
|  |  | rev TACTCTCCTTCAGAACCTGCTCA |
| *Irgm1* | NM_008326.1 | fwd CCGGAGGACAGCAACGTTTT |
|  |  | rev ATACTCCTCAAACCCTGATCCA |
| *Iigp1* | NM_001146275.1 | fwd GATAAAGCAGGGGTGGGTCT |
|  |  | rev CAGGCAAGTGTGCATCAGAAA |
| *Irgm2* | NM_019440.3 | fwd TAAGGCTTCTGAGCAGGTTGC |
|  |  | rev TGGGAATGAATACAGCGTCGG |
| *Gpx1* | NM_008160 | fwd CAGGAGAATGGCAAGAATGA |
|  |  | rev GAAGGTAAAGAGCGGGTGAG |
| *Gpx2* | NM_030677 | fwd ATCAAACGGCTCCTCAAAGT |
|  |  | rev GGGACGATATTCAGGGAATG |
| *Gpx4* | NM_008162 | fwd GCAGGAGCCAGGAAGTAATC |
|  |  | rev GGCTGGACTTTCATCCATTT |
| *Sepw1* | NM_009156 | fwd TAGAGGCAGGGTCCTGAAAG |
|  |  | rev AATCCATCTCTGGCCTGACT |
| *Txnrd1* | NM_015762 | fwd CTACAGACCATTGCCTTGCT |
|  |  | rev ACCTCCTACCCACAAGATCC |
| *Sepp1* | NM_009155.3 | fwd ATCTTGGCAGCAGTAAGCCT |
|  |  | rev TCACTTGCTGTGGTGTCTCA |
| *Sephs2* | NM_009266 | fwd GATAGTGCCGTGGTAGGAGA |
|  |  | rev CTCTGGAAACCACCATCTTG |


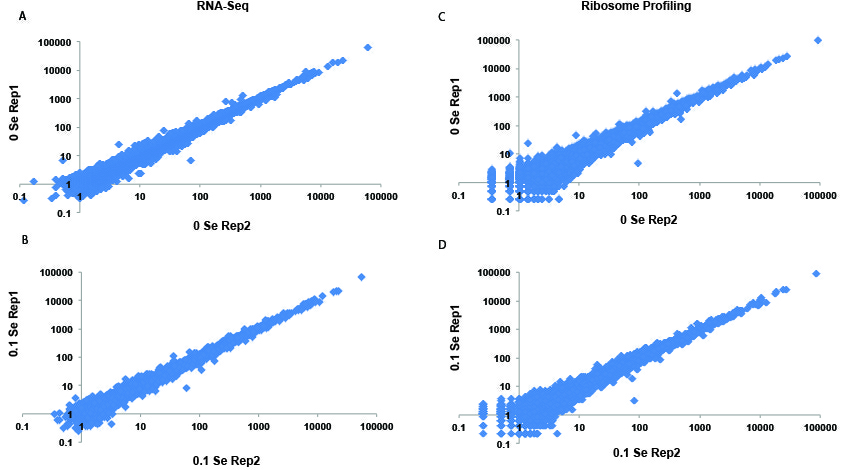


**Figure S1.** Correlation of biological replicates for RNA-Seq and ribosome profiling. Normalized mRNA CPM values (counts per million) generated by edgeR analysis were plotted on the X- and Y-axis for the 0 and 0.1 dietary Se groups for RNA-Seq (**A**) and (**B**) and ribosome profiling (**C)** and (**D**). *R*^2^ values were >0.99 for each biological group.


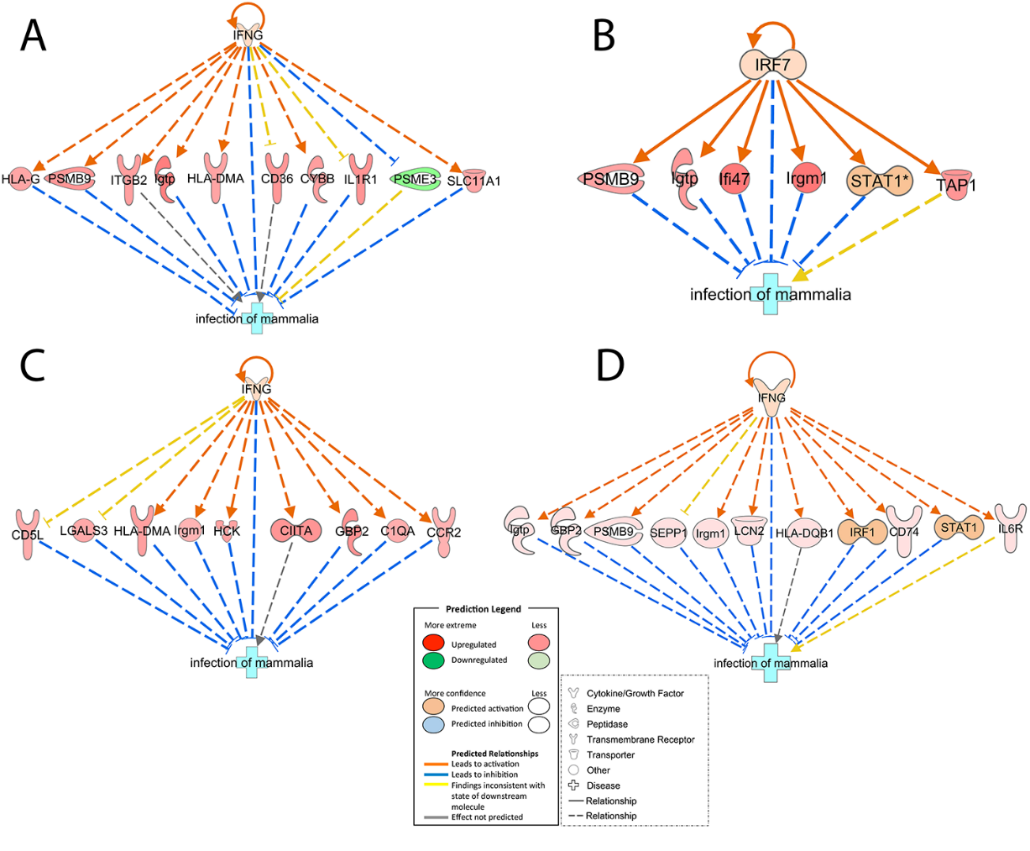


**Figure S2.** Interferon-γ-associated IPA Top Regulator Effect Networks in mice maintained on adequate Se diets compared to those maintained on deficient Se diets. IPA graphs are based on significant (*p* < 0.05) gene expression changes in (**A**) microarray analyses of liver tissue; (**B**) microarray analyses of lung tissue; (**C**) liver RNA-Seq analyses, and (**D**) liver ribosomal profiling analyses.
